# Narratives in European debate concerning new genomic techniques

**DOI:** 10.1007/s11248-024-00416-7

**Published:** 2024-12-04

**Authors:** Marcin Napiórkowski, Andrzej Nowak, Mikołaj Biesaga, Szymon Talaga, Erika Staël von Holstein

**Affiliations:** 1 University of Warsaw, Warsaw, Poland; 2 Florida Atlantic University, Boca Raton, USA; 3 Re-Imagine Europa, Brussels, Belgium

**Keywords:** GMO, NGT, Genome editing, Narratives, Debate, Deliberative democracy

## Abstract

Given the complexity of agricultural problems, it is essential to develop acceptable solutions for various stakeholders with diverse knowledge, viewpoints, and preferences. However, European public opinion has become highly polarized, making constructive discussions on these issues difficult. We present the results of the narrative analysis of media debate on new genomic techniques. The study identified two primary narrative groups: ‘precaution-focused’ and ‘innovation-focused.’ The former emphasizes caution, potential risks, and the need for stringent regulation, while the latter highlights benefits, progress, and the promise of genome editing for sustainable agricultural practices. Within each group of narratives, several distinct narratives were identified. The research has revealed that despite the high polarization, the narratives shared important values and beliefs. Going beyond the dividing narratives and concentrating on common values can depolarize the debate and set the stage for new narratives, enabling constructive debate, concentrating on solving problems, and maximizing collective outcomes.

## Introduction

The accelerating changes to our natural environment, economic instability, and swift societal transformations have created new challenges for food systems worldwide (Béné et al. 2019; Willet et al. 2019; Ben Hassen and El Bilali 2022). To meet these challenges, we need urgent action (Campbell et al. 2018; Halonen 2021) and new regulatory frameworks to guide it (Galli et al. 2020). The European Commission has acknowledged this through the Farm to Fork Strategy’s role in furthering the goals of the European Green Deal. However, continent-wide farmers’ protests contesting European agriculture demonstrate that to meet the challenges of globally changing food systems, we need regulations consistent with scientific knowledge and acceptable to various stakeholders with distinct knowledge, viewpoints, and preferences. The most brilliant innovations will be futile if society rejects them.

This paper is neither the time nor the place to comprehensively review the now historical discourse on the use of Genetically Modified Organisms (GMOs) in the European Union, except inasmuch, narratives from the past can still have a lingering impact and inform the debates today related to, for example, new genomic techniques (NGTs), This paper addresses those narratives in this context. It is important to note that the earlier GMO debate originating in the late 1980s—mid 1990s, arose from the development and implementation of two of the earliest regulatory initiatives in the European Union. These debates became dominated mainly by the precautionary attitudes of the public towards genetically modified plants. As the debate on GMOs intensified, the opinions on GMOs became increasingly polarized, with the public expressing mainly negative sentiments with few advocates of GMOs outside of those stakeholders influenced by science and innovation. The negative reaction to GMOs was motivated by the fears of negative long-term environmental and health consequences related to the use of GMO products, together with a distrust towards industry, big agribusiness and regulatory institutions, as well as the perception of low transparency in the legislative process. There was a widespread perception that GMOs benefit mainly industry and big agribusiness, whilst the benefits to consumers are unclear. Ethical considerations and European culture also played a role, placing a high value on nature and the perception that GMOs violate the rules of nature. The concerns with biosafety in the early 90ties were also amplified by the BSF and HIV epidemics. This was further amplified by consumer organizations and media, which concentrated on the potential dangers of this technology (Bonny 2003; Frewer et al. 2004, Hansesn et al. 2018).

Using new genomic techniques (NGTs) to achieve more agriculturally desirable plants is still a polarizing issue as Carter et al. 2021 observes. To understand the polarization better, in this paper, we have identified and analysed dominant narratives that shape the debate on NGTs and determine public acceptance or rejection of proposed regulatory solutions. We show that although narratives may be categorised into two opposite categories: precaution-focused and innovation-focused, several distinct narratives can be identified. Each of these narratives frames genetic modification differently, is based on different values, and implies different goals. Knowledge of narratives on gene editing may be used to better communicate science and to depolarize the discussion on the topic by identifying the common values of individuals with opposite attitudes toward gene editing in plants. Knowledge of narratives also allows the discussants to use narratives as tools of listening and a better understanding of the concerns and arguments of the other side. Finally, knowledge of polarizing narratives can help us move beyond the polarization of old narratives and create new narratives that foster constructive dialogue in the debate on the genetic modification of plants.

The philosophy of law postulates that law and society co-evolve analogously to the structure of a double helix (Pound 1908; Ruhl 1996; Katz et al. 2020). In this feedback loop, legislation continually shapes society while societal dynamics, its ever-evolving principles, and values concurrently shape legal frameworks. To cope with the changing reality, we must address both sides of this helix, considering the legislative aspects, values, principles, social norms, and collective imagination.

One of the most important challenges in the regulatory process is integrating two seemingly contradictory approaches: evidence-based policymaking and deliberative democracy. Evidence-based policymaking prioritizes scientific research, data analysis, and expert judgment as the primary sources of information for regulation (Cairney 2016). This approach often emphasizes efficiency, objectivity, and technical rigor. However, it has been criticized for oversimplification of policymaking. For example, Greenhalgh and Russell (2009, p. 304) argue that it is “inherently unable to explore the complex, context-dependent, and value-laden way in which competing options are negotiated by individuals and interest groups.” The main principle of deliberative democracy is that regulations should result from a public inclusive and reasoned debate (Gutmann and Thompson 2000; Ryfe 2005; Parkinson and Mansbridge 2012). This approach concentrates on the process leading to regulatory decisions rather than the outcome. Essential for this process are inclusion, diverse perspectives, and mutual understanding. In this view, regulatory decisions should be based on the collective construction of knowledge and solutions (Bächtiger et al. 2018; Cooke 2018).

One of these approaches concentrates on the process, and the other on the outcome, so they can be integrated by basing the deliberation on scientifically established knowledge. It is important to note that science applies only to a relatively narrow range of beliefs, and only some statements can be verified by the scientific method. ﻿The scientific notion of truth applies to empirical knowledge claims based on observation, experimentation, and logical reasoning. Scientific truth claims are typically expressed as theories, hypotheses, laws, or models that aim to explain and predict natural, psychological, and social phenomena. Moreover, truth is not an absolute or eternal concept in science but rather a provisional one that is subject to revision and falsification in light of new evidence or arguments.

The scientific method cannot verify many claims, although they are crucial for society’s functioning. For example, the scientific criterion of truth cannot be applied to preferences, values, norms, aesthetic judgments, ethical judgments, or spiritual claims as they are not subject to empirical testing or logical proof. Arguments have also been made that although facts established by science are objective, their meaning and interpretation, to some degree, involve social, cultural, and ethical elements (Gorski 2013).

Diversity of interests, values, perspectives, and beliefs is the foundation of deliberative democracy, which offers tools to make decisions based on various groups’ views and interests. Public deliberation can, however, be very difficult in a strongly polarized society because the participants may concentrate on attacking those who have opposing views rather than discussing solutions that can maximize a common outcome. As a result, in a highly polarized society, progress on any problem may be blocked, and effectively, everyone loses. Public discussion can depolarize positions on the issue if it follows the rules of deliberative democracy; however, it can have polarizing effects when it does not adhere to the deliberative democracy framework (Caluwaerts et al. 2023). In an ideal epistemic scenario, deliberation and decision-making processes should be thoroughly informed by objective facts, sound logical reasoning, and a diverse range of perspectives (Kuyper 2015). However, constructive deliberation is difficult and may lead to polarization when the issues are complex and difficult to understand, as is often the case in modern societies (Gunn 2017; Bächtiger et al. 2018).

The narrative approach provides a promising angle for tackling complexity in public debates since narratives naturally organize collective reflection and actions (Belinski and Kinder 2006). Social psychology research indicates that narratives are essential for resilience when navigating the complexities of our world and adapting to change (Sools and Mooren 2012). Narratives help individuals make sense of the world (Jones and Song 2014). We use stories to comprehend reality, stay informed, communicate with others, and express values (Hammack and Toolis 2016). Different groups construct unique narratives reflecting various perspectives and preferences, especially in times of complexity and uncertainty. These narratives shape policy development, regulatory choices, and scientific and legal discourse (Caudill 2002). The narrative approach is thus one of the most effective methods for summarising the public debate and involving the generating public in deliberation (Chen and Burgess 2021).

It is vital to note that in social sciences, narratives are not inherently opposed to facts; they serve as essential tools for conveying information, explaining reality, and fostering community. Using narratives to communicate science allows policymakers to overcome two major communication barriers: the knowledge fallacy and the empathy fallacy (Crow and Jones 2018). The authors argue that policymakers usually assume that individuals are knowledgeable and engage in the cognitive processing of rational arguments; however, they are usually emotion-driven and respond to communication intuitively, so rational arguments often fall into a vacuum. The alternative approach, intended to target emotions, assumes that authentic stories can influence audiences through shared human empathy evoked by emotive narratives. However, responses to such emotional appeals are influenced by individual biases, which may result in reactions that are not as consistent or predictable as assumed.

In the context of genome editing, narratives can play a crucial role by incorporating diverse perspectives that go beyond expert opinions. Storytelling thus enables the inclusion of non-expert, non-dominant views in the deliberation, such as based on a relationship with nature (Burger and Chen 2021). Concerns about new genomic techniques can be divided into intrinsic and extrinsic concerns (Carter et al 2021). According to the authors, the science community is less willing to engage in a dialog with more skeptical audiences who express intrinsic concerns such as “Playing with nature” or “Violating the rules of God.” However, the results of a questionnaire study conducted on over four thousand participants suggest that concerns about these narratives often convey valuable insights into people’s risk assessments and can lead to fruitful dialogue with science and progress-oriented biologists. A similar argument was put forward by Almeida and Ranisch (2022), who argue that when engaging the public in ethical discussions about heritable genome editing, it is essential to go beyond practical concerns and consider the deeper categorical and sociopolitical implications.

As often happens in multidimensional discussions involving many intertwined issues, conflicting sides of a polarized debate may share many values and goals. However, the commonalities remain hidden behind highly polarized narratives that describe the world as enemies and threats rather than challenges, problems, and compromises. In this paper, we will examine how we can use a better understanding of the narratives in the debate about genomic techniques to explore the values and principles underlying different positions in the debate and depolarize it.

## Method

Similarly to Biesaga et al. (2023), the authors used a mixed-method approach to analyze a large corpus of texts. The authors used advanced data-mining and Natural Language Processing tools and supplemented them with qualitative analysis of selected texts to explore the subtle balance between the top-down production of narratives by selected European news outlets and their bottom-up reception in social media.

Our goal was to identify the main narratives in Europe concerning genetic modifications of plants. We have used an existing dataset of texts we have been collecting since 2017 from European news outlets published in English and filtered it using keywords to identify the papers relevant to the topics of interest. Because this dataset is not a random sample of publications on the topic, we have decided to skip the quantitative statistics. The articles were collected from 10 leading European news outlets publishing in English. Specifically, they included Daily Express, Daily Mirror, EURACTIV, EUobserver, Euronews English, POLITICO Europe, The Guardian, The Parliament Magazine, Sputnik, and Russia Today (see Table 1.). This choice enabled us to create a large, linguistically homogeneous corpus covering all of Europe and including a wide range of sources from scientific and political communications to potential sources of disinformation. At the same time, such selection of sources does not allow us to conduct a detailed geographic analysis and identify differences between regions, but the narrative map we have created can be used for further discourse studies that take into account geographic variation, and potentially for surveys or focus groups that would show differences between different social groups or generations.

**Table 1:** The number of articles in the overall corpus and filtered corpus by outlet

News outlet	Number of articles	Number of articles filtered by keywords
Daily Express	53,836	1029
Daily Mirror	114,505	1614
EURACTIV	8506	1131
EUobserver	9434	299
Euronews English	32,341	949
POLITICO Europe	11,350	544
The Guardian	65,901	2555
The Parliament Magazine	1369	149
Sputnik News	44,290	821
Russia Today	31,287	754

The goal of our analyses was to identify the main narratives in the discussion on the genetic modification of plants. We make no claims concerning the relative popularity of the identified narratives.

We started by collecting all posts created by those journals on Facebook from 1 January 2017 to 25 October 2020. In addition to the textual content, for each post, we gathered quantitative metrics reflecting the social media engagement for each post, encapsulated within the Interactivity Index (INI). This index, computed as a weighted average of likes, comments, and shares, served as a proxy for the relative popularity and engagement levels elicited by individual posts across the spectrum of outlets.

For each post, we followed the link to a promoted article on the webpage of a given outlet and scraped its content. Through this approach, we aggregated a comprehensive database comprising 372,819 articles. To focus our analysis on articles pertinent to the discourse surrounding genetically modified organisms (GMOs) and new genomic techniques (NGTs), we selected articles containing at least one of the following keywords: “gene”, “gmo”, “agriculture”, “glyphosate”, “dna”, “genome”, and “Monsanto”. We selected these keywords based on the pilot unsystematic qualitative research in media outlets and social media. Consequently, this filtering process gave us a refined thematic subset comprising 9,845 articles for further examination.

Within the refined corpus consisting of 9,845 articles, we conducted topic modeling utilizing the Latent Dirichlet Allocation (LDA) algorithm (Blei et al. 2003). We used a full-featured, industrial-strength implementation provided by the Gensim library for Python programming language (Rehurek and Sojka 2010). It allowed for the identification and segregation of distinct thematic clusters (topics) that were characterized by similar word distribution.

The LDA method assumes that a text is a bag of words. The observed patterns in the document stem from the blend of several underlying topics characterized by a unique probability distribution over words. Consequently, documents sharing common themes are expected to exhibit similarity in their lexical compositions. While LDA represents a somewhat simplistic approach that does not explicitly consider higher-order dependencies between words (such as bigrams, trigrams, etc.), it usually produces easily interpretable results.

While extracting topics through Latent Dirichlet Allocation (LDA) is fundamentally data-driven, the critical task of determining the optimal number of topics requires the researcher’s decision. We employed a coherence measure advocated by Röder et al. (2015), which evaluates both intra-topic coherence and inter-topic diversity. This method required computing multiple LDA models across varying numbers of clusters (topics). Specifically, we tested solutions in the range of 8–20 clusters, ultimately selecting the configuration that maximized the UMass coherence index proposed by Mimno et al. (2011). Finally, we converged on a partitioning into 20 topics.

The topic classification provided by LDA operates within a probabilistic framework, wherein each article is associated with the probability of covering any given topic. Consequently, in some cases, when the likelihoods of belonging to a given topic are similar, assigning the article to a single topic may be somewhat arbitrary. However, this issue affects any probabilistic topic-modeling method and, therefore can hardly be avoided. Moreover, it underscores the multifaceted nature of articles, which frequently discuss multiple topics and issues simultaneously.

For each article, we had two measures – its fit to a given topic and its interactivity index. Fitness describes how typical or representative this article is for the topic. Interactivity is based on the social media reactions to the paper, as described above. For each topic, 20 articles with the highest fitness and 20 with the highest interactivity were provided to interpret which articles had the most influence on the readers and which were most typical for a given topic.

This material (sets of 20 + 20 articles from each of 20 different topics) was then subjected to qualitative analysis. The aim was to move from subject-driven *topics* (i.e., determined by what is being written about) to action-driven *narratives* based on identification of patterns of change (e.g., decline, progress, stagnation, crisis, recovery) determined by what is going on or how things change in time.

We followed a path similar to the work of Vladimir Propp (1968), who used lexicons and catalogs of fairy tales to build a new narrative classification of folk tales (Meletinsky 1974). Similarly, we used machine-extracted topics that served a function analogous to "motifs" or "tropes." To identify the narratives, we have focused on three main dimensions.

First, following the structural method of narrative analysis (Barthes 1975), we clustered the *motives* identified by topic analysis according to the *function* they performed. For example, emerging references to the movie Jurassic Park (dinosaurs escaping from cages) served the same role as descriptions of pollen drift. Dinosaurs were simply larger and easier to present as a symbol of "technology escaping scientists."

The next step was to find recurring *structures* associated with changes in certain values over time. Is the situation worsening or improving, or perhaps we are on the threshold of a grand challenge that will change the trajectory? The number of such structures turned out to be significantly smaller than the number of clustered motifs, making it possible to further combine the narratives into larger, more general structures.

The final step was to isolate the *narrative communities*, that is, the "we" and "they" representations implicitly and explicitly contained in the narratives. Who is the object of concern, and who is the enemy? To whom and about whom do the narratives speak?

After delimiting the narratives based on lexical analysis, we have also analysed typical visual representations, creating unique iconographies for each narrative.

## Results

As a result of this process, a map of narratives was created, including two main narrative stances (innovation-oriented and precaution-oriented) and 8 narratives.

**Table:** 

Precaution-oriented narratives	Innovation-oriented narratives
Unpredicted consequences	Progress
Violating the rules of nature	We need to face a crisis
Greed destroys the traditional way of life	The suspicions have been addressed and tested
We have heard these promises before	We have more precise technologies than ever

### Narrative 1. Unpredicted consequences

This narrative expresses fear that the real consequences of “messing up” with nature may not be predicted. It highlights the complexity of reality and emphasizes long-term consequences over short-term benefits. It critiques an allegedly narrow approach taken by some academics and companies.

This narrative features a consistent visual language depicting contamination. Commonly used images include expansive fields under gloomy skies (without sunlight), symbols of uncontrolled proliferation like milkweed, deceased or weakened insects, and anti-contamination suits.

Numerous texts and online discussions draw parallels to the Jurassic Park franchise. The storyline revolves around unnaturally recreating long-extinct creatures, symbolizing humanity’s hubris in overcoming natural boundaries. Despite safety measures, containment proves impossible, as illustrated by the quote: "Your scientists were so preoccupied with whether or not they could, they didn’t stop to think if they should." This captures a common concern about technology and its relationship with nature.

### Narrative 2. Violating the rules of nature

This storyline revolves around immutable principles. It presents nature and technology as inherently opposite domains. Boundaries between species were created by nature. They drive the right pace and trajectory of evolution.

This narrative employs a visual lexicon highlighting the notion of monstrosity. Traditional symbols of nature, such as apples, are either marred by ubiquitous syringes or metamorphosed into something peculiar. Images of monstrous hybrids, such as an apple resembling a frog or a tomato with tentacles, represent transgressing natural boundaries. Creating new life forms represents humanity’s ultimate hubris.

Depictions in various media frequently allude to Mary Shelley’s Frankenstein or its film adaptations, labeling genetically modified organisms as "Frankenfood." Genetic engineering paves the way for transformations not only in plants and animals but also in humans. As we proceed down this path, species boundaries may blur, potentially dividing one species into many – giving rise to a new race of "superhumans" and further exacerbating social inequality between rich and poor. This perspective aligns with anti-egalitarian views on biotechnology.

### Narrative 3. Greed destroys a traditional way of life

The narrative illustrates a one-way path from local farming built on harmony with nature to its industrialized version built on exploiting nature and people. The discourse on genome editing becomes linked to a broader backlash against the industrialization and commercialization of our daily lives, incorporating anti-globalization arguments and general critiques of modern neoliberal capitalism driven by unchecked greed.

The past is valued positively (nostalgia), while the present and possible future are pictured as negative. Bottom-up, grassroots initiatives are valued positively, while the current industrialized model is associated with top-down policies and concentration of wealth.

Introducing genetically engineered crops undermines traditional values by intensifying competition, favoring big business while eroding the traditional coexistence model in rural communities. Biotechnology is claimed to strengthen corporate control over agriculture. Various products and solutions are portrayed as "Trojan horses" for large industries to penetrate small farming, seize control, and dominate it. This raises questions of ownership ("no food shall be grown that we do not own") and intellectual property rights. In extreme versions of this narrative, new technologies contribute to farmer suicides by trapping them in a spiral of debt and despair. Patenting seeds is depicted as the epitome of greed, leading to hunger, despair, and, ultimately, farmer suicides. The term "seed slavery" symbolizes a new form of radical dependency resulting in extreme poverty.

### Narrative 4. We have heard these promises before

This narrative accuses scientists and industry of overpromising and overselling (see Bresanini, Mautino 2014). It engages with the conversation surrounding the issue rather than focusing solely on the problem itself.

In public discourse, GMOs and genome editing technologies have often been presented as “ultimate solutions” to humanity’s biggest challenges. This narrative is built on disillusionment.

In the visual form, this narrative typically employs vector graphics instead of photographs, aligning with its more abstract and discursive nature. The visuals often depict self-assured scientists manipulating DNA (commonly represented as a ladder), symbolizing their perceived control over genetic material and arguments to sway public opinion. Notably, several of these images are derived from positive communications promoting novel genome editing technologies’ potential benefits.

### Narrative 5. Progress

This innovation-driven narrative expresses the idea of progress. It stems from the Enlightenment, optimism, and utopianism. It takes a global perspective rather than focusing on local aspects of technologies. Nature and technology are distinctly positioned on a continuum leading from a "difficult past" to a "promising future".

This narrative is frequently depicted through conventional lexical and visual metaphors of progress and transformation: images depicting triumph, sunny landscapes, collaborative individuals, and joyful farmers, often from the Global South. Vector graphics, commonly in the form of diagrams and infographics, are more prevalent than in precaution-focused narratives. The primary color is spring green, symbolizing hope and prosperity.

A futuristic Augmented Reality interface, reminiscent of those seen in science fiction films like "Minority Report” is often employed to represent (non-ironically) the idea of control and manipulating reality.

### Narrative 6. We need to face a crisis

In contrast to the optimistic (and quite deterministic) story of progress, this narrative is built around the notion of a challenge. The narrative addresses topics such as climate change, inequalities, and migration. What we have achieved in past centuries could be jeopardized if we fail to adapt. This argument is exemplified by the image of diverging paths, emphasizing the crucial juncture of decision-making. This narrative strongly underscores the negative consequences of not adopting specific solutions.

Visual elements in this narrative often allude to diverging paths, using imagery such as crossroads, board games, or "either-or" scenarios depicted on split screens. Potential negative consequences of neglect are frequently portrayed through visuals like scorched earth and starving individuals.

This narrative is often culturally mediated by metaphors borrowed from "The Armageddon" and other Hollywood films where a catastrophe is preventable through collective effort.

### Narrative 7. The suspicions have been addressed and tested

This narrative is compelling due to its metadiscursive nature. It focuses on fostering open dialogue between experts and the general public regarding genome editing. Emphasizing transparency, trust, and constructive debate as essential values in addressing concerns related to this technology encourages a more inclusive approach that considers various perspectives before making informed decisions based on empirical evidence.

Genome editing is presented as something old and well-established. The extensive history and proven safety record of various genome editing techniques are highlighted. By acknowledging criticisms and addressing concerns through rigorous testing and evaluation, these narratives stress the reliability and integration of such innovations into daily life without any extreme side effects, as initially feared by skeptics.

### Narrative 8. We have more precise technologies than ever

This narrative also focuses on the long history of modifying living organisms for agricultural purposes. It presents new genome editing technologies as refined versions of ancient techniques used both in farming and in nature.

This narrative emphasizes the difference between “old” GMOs and “new” genome editing technologies, emphasizing qualitative differences and highlighting distinctions such as lack of transfer of genetic material between species.

This subnarrative familiarizes genome editing technologies by drawing parallels to traditional practices like selective breeding, crossbreeding, and other methods used for centuries in farming. It employs positive associations with everyday objects such as seedless fruits or popular dog breeds to challenge the notion of "natural" variations versus "technologized" GMOs.

## Discussion

Our analysis shows that the debate around new genome editing technologies is more complicated than a simple division into "pro-NGT" and "anti-NGT" would indicate. While, in general, the debate can be divided into innovation-focused and precaution-focused narratives, there are several significantly different narratives operating within each camp.

Each of the identified narratives focuses on different key topical issues currently being debated in the EU with regard to NGTs. (It is worth noting that the relation also works the other way – focusing on any of these topical issues increases the chance of triggering a particular narrative). For example, narrative 1 (Unpredicted consequences) mentions the dangers of cross pollination or bee or butterfly extinction. Narrative 2 gives much more attention to possible harmful consequences for consumers. Narrative 3 (Greed destroys the traditional way of life) devotes much attention to the issue of patenting and intellectual property. Narratives 2 and 4 stress the continuity between GMOs and NGTs. Similar diversity can be observed in the innovation-focused camp with Narrative 5 focusing on fighting hunger and malnutrition, Narrative 6 devoting a lot of attention to climate change adaptation and mitigation, and Narratives 7 and 8 interested in differences between old and new technologies. The tension between the desire for progress and the desire to preserve traditional rural life can be particularly pertinent in “new EU” countries such as Poland, as the recent protests by farmers against the European Green Deal have shown.

Innovation-oriented narratives seem less concerned with the cultural and social dimensions of rural life. Conversely, the economic dimension is presented "from a bird’s eye view," described by general factors (higher yields, lower losses) rather than individual human fates. This may be an important clue for scientists and science communicators, what to include in their message to create a less polarising debate, because currently pro-innovation narratives are often perceived as less empathetic or even dehumanising.

This is also the result of the fact that the two main camps communicate in different languages. Innovation-oriented communication is much more general and abstract. Visually, it is often based on vector graphics and diagrams. This can make it seem more "cold" and distant from the problems of ordinary people. Precaution-oriented communication more often depicts human stories. It is more firmly rooted in locality and appeals more strongly to emotions. It is eager to use strong visual metaphors related to monstrosity, dystopia, or enslavement.

Both sides accuse each other of arrogance, not listening, and spreading false information (this is especially visible in narratives 4 and 7). The debate around NGTs is heavily dependent on its history.. It follows that there is space and need for the construction of new "metanarratives" about dialogue and joint problem-solving.

In many areas, the dispute is not really about NGTs. New technologies only serve as a "prop" to address deep fears and hopes. Without addressing these challenges, we will not have socially acceptable regulations. So, the problem is not only about evidence-based policies but also about building such a framework for deliberative democracy so that these policies better address the values and interests of different groups.

Narrative analysis clearly shows that to build an acceptable regulatory system, we need to build a dialogue based on shared values and interests. For example both camps mention sustainability and climate change prevention as important goals. Many analysed texts from both sides also tend to mention generational issues, from very general, such as saving the Earth for our children, to more specific, like avoiding dangerous substances in food targeted for children or making farming a more attractive way of life for future generations of rural citizens. These values may be a good starting point for depolarizing the debate and building potential narrative bridges.

## Conclusions

Creating a value-neutral map of existing narratives can help understand the social dynamics of the conflict. We tend to think of narratives as a tool of persuasion – from the point of view of the sender. However, in the context of conflict management, narratives are, first and foremost, an effective tool for listening to others. Refocusing on thinking in terms of narratives instead of immediately analyzing and refuting arguments may allow us to understand the values, goals, and beliefs of others.

Then, in the next step, narratives may indeed prove to be an effective tool of communication. We may use them to build bridges between data, interpretation, and action or to put multiple dimensions of an issue together meaningfully. As a result, better narratives pave the way for better and more socially acceptable policies. However, these next steps are only possible if we start with listening. Before we start advocating for particular solutions, we need to focus on the shape of the debate. Reframe the issue and take the initiative.

By analyzing the narratives people use, we can understand what is really important for them, why they use certain frames of reference, and the way they view the world. We can understand the true goals and values behind positions. This is especially important in highly polarised issues like genome editing.

By addressing the motives, goals, and values in a dialog instead of attacking positions, we can break the stalemate that makes finding solutions nearly impossible. One of the best strategies for overcoming the stalemate is to start a constructive dialogue on how to achieve common goals and maximize common gains. To do so, it is essential to adopt a problem-solving attitude. In this approach, we should help the partners solve their problems in dialogue.

In a dialogue, especially with policymakers, adopting the perspective of the other side allows us to understand their limitations that may impact their decision-making process. In these instances, active listening and integration foster a spirit of cooperation, which is more productive than attempting to persuade. Avoiding defensiveness is another key aspect of productive dialogues; negation merely echoes the arguments presented by opponents.

There might be situations where policymakers find themselves at odds with their constituents’ opinions, even if these views contradict their personal beliefs. In such cases, demonstrating how to present issues effectively to voters through compelling narratives can prove invaluable. By crafting persuasive stories that resonate with the public, we help policy-makers navigate complex situations and maintain support from their voter base.

## Data Availability

No datasets were generated or analysed during the current study.
